# Identification of Entacapone as a Novel β-Arrestin 1 Biased Antagonist of CXCR7

**DOI:** 10.3390/molecules31152606

**Published:** 2026-07-26

**Authors:** Liangrui Shi, Yan Huang, Lian Li, Huan Li, Xin Li, Zenghao Bi, Junke Liu, Sanyin Zhang, Zhaotong Cong, Bojun Wang, Shilin Chen

**Affiliations:** 1 College of Pharmaceutical Sciences, Guizhou University of Traditional Chinese Medicine, Guiyang 550025, China; 2 Institute of Herbgenomics, Chengdu University of Traditional Chinese Medicine, Chengdu 611137, China; 3 School of Basic Medical Sciences, Chengdu University of Traditional Chinese Medicine, Chengdu 611137, China; 4 School of Pharmacy, Nanjing University of Chinese Medicine, Nanjing 210023, China

**Keywords:** CXCR7, entacapone, MST, β-arrestin signal, molecular docking

## Abstract

Background: The C-X-C chemokine receptor type 7 (CXCR7), also known as atypical chemokine receptor 3 (ACKR3), primarily signals through β-arrestin and plays a pivotal role in tumor progression, inflammation, and neurodegenerative diseases. CXCR7 antagonists block chemokine binding and dampen β-arrestin signaling. Accordingly, identification of such antagonists is highly desirable for therapeutic development against CXCR7 driven pathologies. Methods: Through microscale thermophoresis (MST) screening of a Food and Drug Administration (FDA)-approved drug library, entacapone was identified as a CXCR7 binder. The NanoBit complementation assay was employed to evaluate the effect of entacapone on CXCR7 mediated β-arrestin 1/2 recruitment. Molecular docking was performed to predict the binding pocket and binding sites. Results: Entacapone specifically bound to CXCR7 with a Kd value of 6.04 µM. Although entacapone did not directly activate CXCR7, it selectively inhibited CXCL12 and VUF11207 induced β-arrestin 1 recruitment with no significant effect on β-arrestin 2 recruitment. Molecular docking suggested that entacapone interacted with key residues via hydrophobic contacts, including Trp100, Phe124, Gln301, and Leu305, and formed hydrogen bonds with Ser103, Asn108, and Tyr51 in the transmembrane core, collectively suggesting a possible binding mode compatible with stabilizing the receptor in an inactive conformation. Conclusions: Entacapone, a clinically well established COMT inhibitor, is reported for the first time as a novel biased antagonist of CXCR7, providing a new candidate molecule for drug repurposing.

## 1. Introduction

G protein-coupled receptors (GPCRs) constitute the largest membrane protein superfamily in humans, orchestrating diverse physiological and pathological processes while serving as the molecular targets for approximately 35% of all approved drugs [1,2]. Despite their well established pharmacological significance, conventional GPCR drug discovery has predominantly prioritized orthosteric agonists or antagonists that indiscriminately engage all downstream signaling outputs. Such a non selective strategy fails to account for the intrinsic signaling diversity of GPCRs and frequently elicits on target adverse effects [3,4]. In this context, C-X-C chemokine receptor type 7 (CXCR7) serves as a prototypical model for investigating GPCR biased signaling [5,6].

Unlike classical chemokine receptors, CXCR7 lacks the canonical DRYLAIV motif in its second intracellular loop, instead featuring a DRYLSIT sequence. This single amino acid substitution disrupts the conserved ionic lock required for Gα protein coupling, rendering CXCR7 incapable of eliciting classical G protein mediated calcium mobilization or cAMP suppression [7,8]. Instead, CXCR7 preferentially signals through β-arrestin, which coordinates receptor internalization, MAPK/ERK pathway activation, and downstream transcriptional regulation [9,10]. Additionally, CXCR7 exhibits pronounced constitutive activity, spontaneously recruiting G protein coupled receptor kinases (GRKs) and sustaining basal β-arrestin engagement in the absence of exogenous ligands [11]. Functioning as a scavenger receptor, CXCR7 internalizes and degrades its endogenous chemokine ligands, CXCL12 and CXCL11, thereby modulating extracellular chemokine gradients and indirectly regulating CXCR4 mediated chemotaxis [12,13]. Pathologically, CXCR7 is aberrantly overexpressed across multiple malignancies, including breast cancer, glioblastoma, and non-small cell lung cancer, where it drives tumor cell migration, angiogenesis, and therapeutic resistance [14,15]. Within the central nervous system, dysregulation of the CXCL12/CXCR4/CXCR7 axis contributes to neuroinflammatory cascades underlying Alzheimer’s disease, multiple sclerosis, and ischemic stroke [16]. Despite its well recognized therapeutic potential, the development of highly selective biased ligands targeting CXCR7 remains limited, presenting a bottleneck for both mechanistic investigation and clinical translation [17].

Recent advancements in CXCR7 pharmacology have led to the identification and characterization of multiple small molecule modulators [18]. Among these, ACT-1004-1239 has emerged as a potent, highly selective, and orally bioavailable CXCR7 antagonist with promising translational potential [17]. Pharmacokinetic evaluations further indicate that the metabolism of ACT-1004-1239 is significantly modulated by the CYP3A4 inhibitor itraconazole, a critical consideration for clinical coadministration strategies [19]. Additionally, VUF16840, a small molecule inverse agonist, effectively suppresses the constitutive activity of CXCR7, offering novel insights into the regulation of its basal signaling [20]. Furthermore, accumulating evidence underscores the therapeutic utility of CXCR7 antagonism in neuroinflammation, notably through the facilitation of demyelinated lesion repair via synergistic immunomodulatory and remyelinating mechanisms [21]. Additionally, VUF11207, a prevalidated small molecule agonist, selectively recruits β-arrestins at CXCR7 without engaging canonical G protein pathways, serving as a critical pharmacological tool for dissecting the receptor’s intrinsic transducer coupling bias [6]. Despite these advancements, the development of CXCR7 antagonists remains challenging. Consequently, there is a pressing need to discover novel biased ligands capable of precisely modulating CXCR7 mediated β-arrestin signaling, thereby maximizing therapeutic efficacy while minimizing adverse effects [22]. Notably, drug repurposing strategies have increasingly identified unexpected GPCR modulatory activities among clinically approved drugs. The present study establishes entacapone, a Food and Drug Administration (FDA) -approved [23] catechol-O-methyltransferase (COMT) inhibitor for Parkinson’s disease [24], as a novel biased antagonist of CXCR7, revealing a previously unrecognized pharmacological activity and highlighting the potential for drug repurposing.

In this study, we employed microscale thermophoresis (MST) to screen an FDA-approved drug library comprising over 2000 structurally diverse small molecules, thus identifying entacapone as a specific binder of CXCR7. Entacapone, a second generation, reversible inhibitor of COMT, was approved in 1999 as an adjuvant therapy for Parkinson’s disease [25]. However, whether entacapone could modulate GPCRs remains to be elucidated. Subsequently, by integrating the NanoBiT complementation reporter system with molecular docking simulations, we systematically characterized the modulatory effects and molecular mechanisms of entacapone on CXCR7, which mediated β-arrestin signaling. Specifically, our investigation focused on (1) quantifying the direct binding affinity of entacapone to CXCR7; (2) evaluating its agonistic or antagonistic efficacy regarding β-arrestin recruitment; and (3) delineating the predicted binding conformation and critical residue interactions. Collectively, this work aims to establish entacapone as a novel biased antagonist of CXCR7, thereby providing a valuable pharmacological tool for dissecting CXCR7 related pathophysiology. These findings lay a foundation for future exploration of entacapone repurposing in oncology, inflammatory conditions, and neurodegenerative disorders. Biased CXCR7 antagonism by entacapone represents a promising direction for future preclinical investigation, pending direct efficacy validation.

## 2. Results

### 2.1. Identifying Entacapone as a CXCR7 Binder

**Figure 1:**
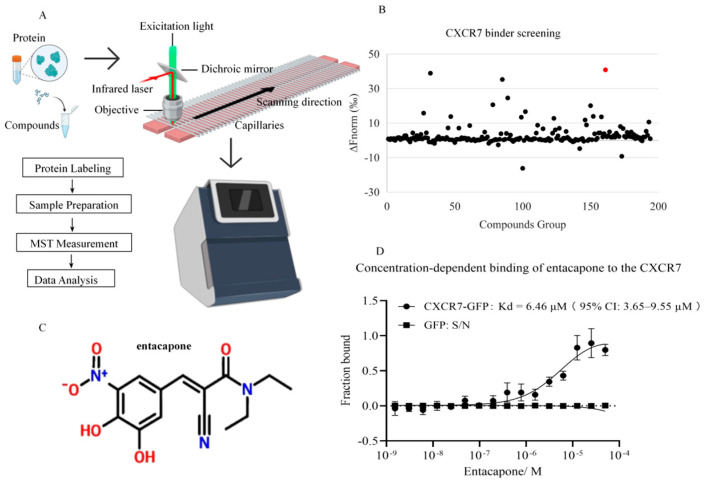
Identification of CXCR7 binding compounds using MST. (**A**) Schematic illustration of the MST experimental workflow for ligand receptor binding analysis. (**B**) Primary screening of the FDA-approved drug library. *n* ≥ 3 independent experiments. (**C**) Chemical structure of entacapone. (**D**) Affinity validation of entacapone binding to CXCR7. *n* ≥ 3 independent experiments.

MST quantifies biomolecular interactions by measuring fluorescence changes in a microscopic temperature gradient [26]: an infrared laser generates localized heating within capillaries containing fluorescently labeled protein and ligand, inducing thermophoretic movement that is detected via fluorescence emission (Figure 1A). The equilibrium dissociation constant (Kd) serves as the metric for binding affinity. To facilitate efficient screening of an FDA-approved drug library, compounds were pooled into approximately 200 groups (each group contains 10 compounds). The pool containing entacapone elicited a normalized fluorescence change significantly exceeding the background threshold (Figure 1B,C), indicating a potential interaction with CXCR7.

Subsequent affinity validation assays (Figure 1D) confirmed that entacapone binds to the CXCR7-GFP fusion protein with typical saturation kinetics. Nonlinear regression analysis yielded a Kd value of 6.46 μM, indicative of direct binding between entacapone and CXCR7. Conversely, entacapone exhibited negligible interaction with GFP alone, characterized by a low signal-to-noise ratio and the absence of a fit-for-purpose binding curve. This control confirms that the interaction is specific to the CXCR7 moiety rather than the GFP tag or nonspecific components within the cell lysate. Collectively, these MST data establish entacapone as a direct binder of CXCR7, providing a solid basis for subsequent functional and mechanistic investigations.

### 2.2. Regulation of CXCR7 Signaling by Entacapone

**Figure 2:**
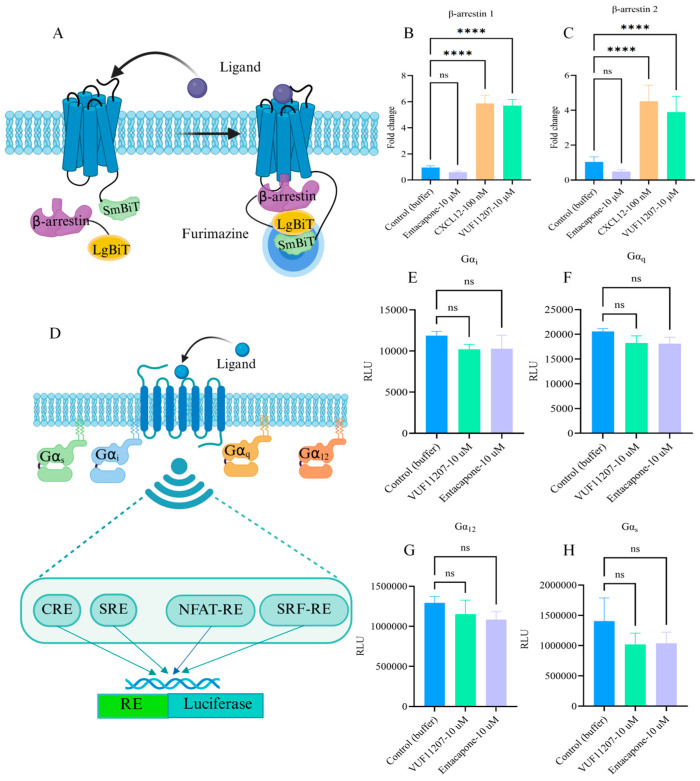
Assessment of entacapone effects on β-arrestin and G protein coupling. (**A**) Schematic illustration of the NanoBiT complementation assay for detecting β-arrestin 1/2 recruitment to CXCR7. (**B**) Entacapone (10 μM) does not induce β-arrestin 1 recruitment compared to vehicle control (ns), while CXCL12 (100 nM) and VUF11207 (10 μM) elicit robust recruitment (**** *p* < 0.0001), *n*  =  3 independent experiments. (**C**) Entacapone (10 μM) does not induce β-arrestin 2 recruitment compared to vehicle control (ns), while CXCL12 (100 nM) and VUF11207 (10 μM) elicit robust recruitment (**** *p* < 0.0001), *n*  =  3 independent experiments. (**D**) Schematic illustration of the reporter gene assay for detecting G protein pathway activation. (**E**–**H**) Entacapone (10 μM) has no significant effect on Gα_i_ (**E**), Gα_q_ (**F**), Gα_12_ (**G**), Gα_s_ (**H**) protein activation compared to vehicle control or the positive control VUF11207 (10 μM), *n*  =  3 independent experiments.

The regulatory effects of entacapone on CXCR7 mediated β-arrestin 1/2 recruitment were systematically evaluated using the NanoBiT complementation reporter system (Figure 2A). Treatment with entacapone (10 μM) failed to induce significant recruitment of either β-arrestin subtype (Figure 2B,C), with luminescence signals remaining comparable to those of the vehicle control. This result indicates that entacapone lacks intrinsic agonistic activity at CXCR7. Conversely, the endogenous ligand CXCL12 (100 nM) and the synthetic agonist VUF11207 (10 μM) elicited robust and statistically significant recruitment of both β-arrestin 1 and β-arrestin 2 (*p* < 0.01), thereby validating the sensitivity and reliability of the assay system.

To determine whether entacapone activates classical G protein pathways downstream of CXCR7, a reporter gene based functional assay was used to systematically evaluate its ability to activate the Gα_i_, Gα_q_, Gα_12_, and Gα_s_ pathways (Figure 2D). As shown in Figure 2E–H, in the Gα_i_ (Figure 2E), Gα_q_ (Figure 2F), Gα_12_ (Figure 2G), and Gα_s_ (Figure 2H) detection systems, neither VUF11207 (10 μM) nor entacapone (10 μM) caused any statistically significant change in relative luciferase activity compared with the solvent control (ns, *p* > 0.05), indicating that neither compound activated any of the G protein pathways.

VUF11207, a known CXCR7 agonist that potently recruits β-arrestins [27], also failed to trigger G protein signaling in our assay. This observation aligns with the well documented atypical nature of CXCR7; the substitution of the canonical DRYLAIV motif with DRYLSIT in the second intracellular loop destabilizes the ionic lock required for Gα coupling, thereby precluding the formation of a functional G protein binding interface [28]. Our data extend this structural paradigm by confirming that entacapone similarly lacks G protein agonistic potential.

### 2.3. Entacapone Inhibits CXCL12 and VUF11207 Induced β-Arrestin 1 Recruitment

**Figure 3:**
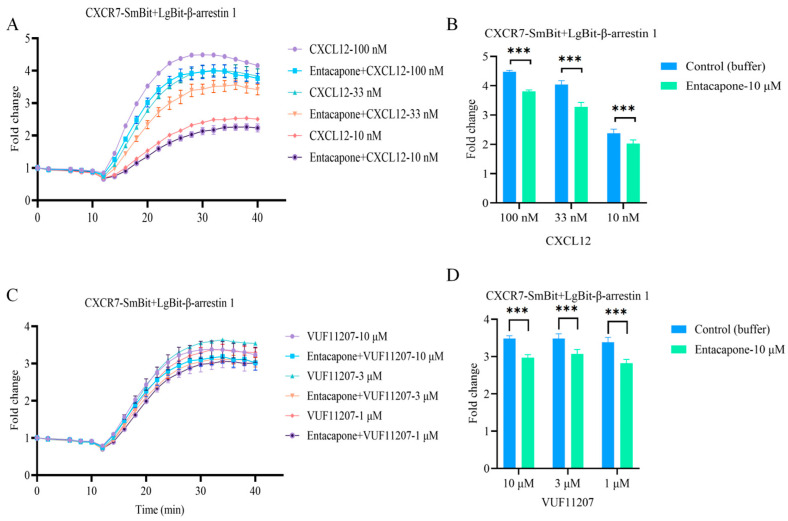
Entacapone inhibits agonist induced β-arrestin 1 recruitment to CXCR7. (**A**) Time course profiles of β-arrestin 1 recruitment induced by a concentration gradient of CXCL12 with or without 10 μM entacapone pretreatment. (**B**) Quantification showing that entacapone effectively inhibits CXCL12 induced recruitment (*** *p* < 0.001 vs. vehicle control; mean ± SEM; *n* = 3 independent experiments). (**C**) Time course profiles of β-arrestin 1 recruitment induced by a concentration gradient of VUF11207 with or without 10 μM entacapone pretreatment. (**D**) Quantification showing that entacapone effectively inhibits VUF11207 induced recruitment (*** *p* < 0.001 vs. vehicle control; mean ± SEM; *n* = 3 independent experiments).

β-arrestin 1 recruitment assay demonstrated that entacapone significantly suppressed β-arrestin 1 recruitment induced by both CXCL12 and VUF11207 in a concentration dependent manner. Time course analysis (Figure 3A) revealed that CXCL12 alone (gradient concentration of 100, 33, and 10 nM) elicited a dose dependent increase in β-arrestin 1 recruitment, reaching maximal levels within 30 min. Pretreatment with 10 μM entacapone markedly attenuated the response across all CXCL12 concentrations, resulting in reduced peak values and a downward shift in the kinetic curves. Endpoint quantification (Figure 3B) confirmed that entacapone significantly inhibited β-arrestin 1 recruitment stimulated by gradient concentrations of CXCL12 (*p* < 0.001). Notably, the inhibitory magnitude remained robust, suggesting effective antagonism even at higher agonist loads.

In parallel experiments using the synthetic agonist VUF11207 (gradient concentrations of 10, 3, and 1 μM), similarly induced concentration dependent β-arrestin 1 recruitment (Figure 3C). Pretreatment with 10 μM entacapone effectively suppressed the β-arrestin 1 response at all tested VUF11207 concentrations. Endpoint quantification (Figure 3D) verified that entacapone significantly inhibited VUF11207 induced recruitment (*p* < 0.001), although the absolute degree of signal suppression appeared distinct from that observed in the CXCL12 assays.

Comparative analysis indicated that entacapone exerted a more potent inhibitory effect on CXCL12 mediated signaling compared to VUF11207, suggesting a preferential sensitivity or differential antagonistic interaction with the endogenous ligand. Collectively, these findings establish that entacapone acts as a potent antagonist of CXCL12 and VUF11207 induced β-arrestin 1 recruitment at CXCR7.

**Figure 4:**
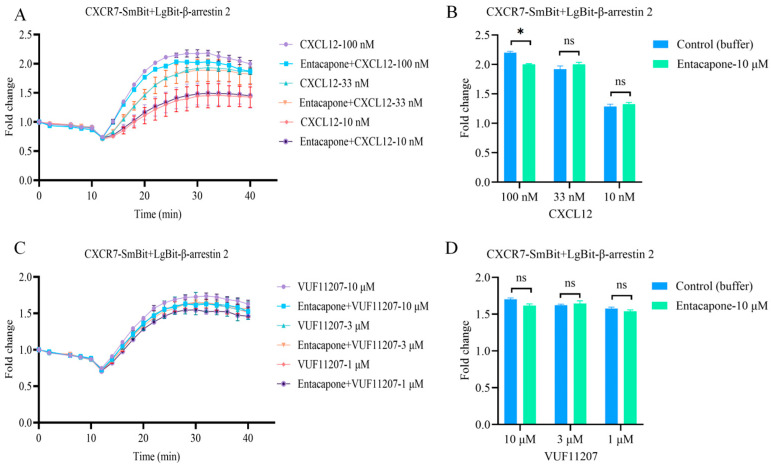
Entacapone exerts no significant regulatory effect on CXCR7 mediated β-arrestin 2 recruitment. (**A**) Time course profiles of β-arrestin 2 recruitment induced by gradient concentrations of CXCL12 with or without 10 μM entacapone pretreatment. (**B**) Quantification showing that entacapone has no significant inhibition (* *p* < 0.05, ns, *p* > 0.05, mean ± SEM; *n* = 3 independent experiments) on CXCL12 mediated β-arrestin 2 recruitment. (**C**) Time course profiles of β-arrestin 2 recruitment induced by gradient concentrations of VUF11207 with or without 10 μM entacapone pretreatment. (**D**) Quantification showing that entacapone has no significant inhibition (ns, *p* > 0.05, mean ± SEM; *n* = 3 independent experiments) on VUF11207 mediated β-arrestin 2 recruitment.

However, the β-arrestin 2 recruitment assays (Figure 4) demonstrated that entacapone failed to significantly modulate CXCR7 mediated β-arrestin 2 recruitment. As shown in the dynamic time course curves (Figure 4A), CXCL12 alone elicited concentration dependent β-arrestin 2 recruitment (gradient concentrations of 100, 33, and 10 nM), with peak responses occurring at approximately 20 min. Pretreatment with 10 μM entacapone produced response curves that virtually overlapped with those of the agonist-alone controls, exhibiting no discernible signal suppression or rightward shift. Quantitative endpoint analysis (Figure 4B) revealed a statistically significant reduction only at 100 nM CXCL12 (*p* < 0.05), whereas no significant differences were observed at 33 nM or 10 nM (*p* > 0.05). Notably, this marginal effect was substantially weaker than the antagonistic activity observed for β-arrestin 1, suggesting that it likely represents experimental variability rather than a biologically meaningful interaction.

Complementary experiments with VUF11207 (Figure 4C) tested whether entacapone modulates β-arrestin 2 recruitment induced by a synthetic CXCR7 agonist. VUF11207 (gradient concentration of 1, 3, 10 μM) alone elicited concentration dependent β-arrestin 2 recruitment. Pretreatment with 10 μM entacapone produced kinetic curves that were visually indistinguishable from those of VUF11207 alone. Endpoint quantification (Figure 4D) confirmed that entacapone did not significantly alter β-arrestin 2 recruitment at any VUF11207 concentration tested (*p* > 0.05).

Collectively, these findings demonstrate that entacapone does not substantially modulate CXCR7 mediated β-arrestin 2 recruitment. This stands in marked contrast to the potent, concentration dependent antagonism observed for the β-arrestin 1 isoform, indicating that entacapone selectively blocks the β-arrestin 1 signaling axis while sparing β-arrestin 2, which is a profile consistent with a β-arrestin 1 biased CXCR7 antagonist.

### 2.4. Binding Mode of Entacapone to CXCR7

**Figure 5:**
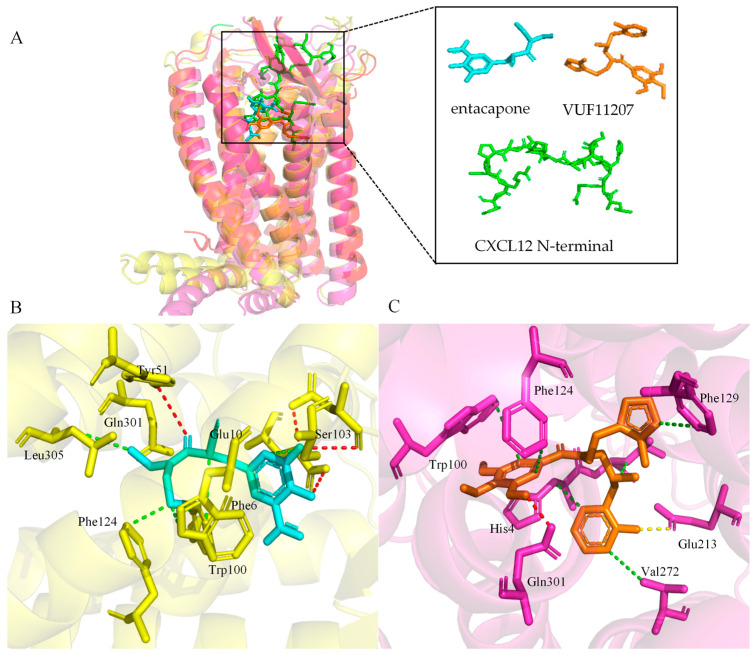
Binding modes of entacapone, VUF11207, and the CXCL12 N-terminal peptide at the CXCR7 orthosteric site. (**A**) Structural overlay of entacapone (cyan, predicted structure), VUF11207 (orange, predicted structure), and the CXCL12 N-terminal peptide (green, PDB ID: 7SK3). All three ligands converge within the transmembrane core but exhibit distinct spatial occupancy patterns. (**B**) Key residue interactions of entacapone with CXCR7. Green dashed lines: hydrophobic interactions, and red dashed lines: hydrogen bonds. (**C**) Key residue interactions of VUF11207 with CXCR7. Green dashed lines: hydrophobic interactions; red dashed lines: hydrogen bonds; and yellow dashed lines: halogen bonds.

To explore the molecular mechanism underlying entacapone’s biased antagonism at CXCR7, molecular docking simulations were performed using AutoDock Vina 1.2.3 for entacapone and VUF11207. As shown in Figure 5A, the two in silico docked small molecules (entacapone and VUF11207), together with the CXCL12 N-terminal peptide extracted from the experimental crystal structure (PDB ID: 7SK3), all occupy the orthosteric binding pocket within the CXCR7 transmembrane core, yet exhibit distinct binding poses and spatial occupancy patterns that may offer a structural basis to rationalize their distinct pharmacological behaviors measured in cellular functional assays. Entacapone inserts into the central cavity with its nitrocatechol pharmacophore oriented toward a hydrophobic subpocket defined by Trp100, Phe124, and Tyr51 in the upper transmembrane region (Figure 5B). In contrast, VUF11207 adopts a more elongated conformation extending toward the extracellular vestibule, while the N-terminal segment of CXCL12 penetrates the transmembrane core with its N-terminal residues establishing multipoint contacts across several helical interfaces (Figure 5C).

Detailed analysis of the entacapone CXCR7 complex reveals that the nitrocatechol moiety forms critical interactions with the receptor core (Figure 5B). The planar catechol ring system is positioned in close proximity to Trp100 and Phe124, while the nitro group and adjacent hydroxyl groups form hydrogen bonds with Ser103, Asn108, Gln10, and Gln301 (Table 1). Additional hydrophobic contacts are contributed by Phe6, Tyr51, and Leu305 (Table 1). This compact binding footprint is concentrated around the central hydrophobic cavity without substantial extension toward extracellular domains. Furthermore, the specific interactions between entacapone and residues Trp100 and Gln301 prompt a hypothetical structural model. In this model, the tight central packing induced by these contacts could potentially restrict the local conformational flexibility of the transmembrane domain, thereby impairing its dynamic rearrangements, which may be essential for proper protein function or allosteric regulation. However, we emphasize that this proposal remains a computational hypothesis derived from static docking simulations.

In contrast, VUF11207 presents a distinctly different interaction pattern (Figure 5C). Its molecular scaffold extends prominently toward the extracellular vestibule, engaging additional residues including Phe129 in the upper transmembrane region and extracellular loop 2. Key interacting residues include Gln301, Val272, and Glu213, which form hydrogen bonds and electrostatic interactions. Additional hydrophobic contacts with Trp100, Phe124, His4, and Leu5 anchor the ligand within the pocket. This extended binding architecture is consistent with the outward tilting of transmembrane helix 6 and the inward movement of transmembrane helix 7, hallmark conformational changes associated with active-state stabilization in class A GPCRs.

Collectively, these structural results provide a structural basis for the pharmacological profiles observed in our functional assays. Entacapone, VUF11207, and CXCL12 compete for the same orthosteric pocket anchored by Trp100, Phe124, and Gln301, enabling entacapone to exert competitive steric antagonism. A critical functional divergence lies in their distinct interactions with Gln301: entacapone engages this residue via hydrophobic stacking, while VUF11207 and CXCL12 rely on hydrogen bonding to stabilize the active CXCR7 conformation. Pre-bound entacapone occupies the polar hydrogen bond acceptor pocket of Gln301, abolishing the essential polar contact required for agonist activation. Even if agonists transiently form hydrophobic contacts with Trp100 and Phe124, loss of Gln301 mediated hydrogen bonding eliminates their capacity to trigger receptor conformational rearrangement. In summary, entacapone competitively displaces CXCL12 and VUF11207 via overlapping hydrophobic interactions with Trp100 and Phe124, disrupts agonist specific Gln301 hydrogen bonds, and rigidifies the CXCR7 transmembrane bundle to lock the receptor in an inactive state.

## 3. Discussion

Entacapone, a second generation reversible inhibitor of COMT, was approved by the FDA on 30 December 1999, as an adjuvant therapy for Parkinson’s disease [24]. Since its clinical introduction, it has been extensively utilized to extend the plasma half-life of levodopa via inhibition of peripheral COMT, thereby mitigating motor fluctuations [29]. Beyond its canonical COMT inhibition, emerging evidence has unveiled additional polypharmacological properties of entacapone, including inhibition of α-synuclein and tau aggregation, suggesting potential neuroprotective efficacy [30], attenuation of ferroptosis in renal ischemia/reperfusion injury, and modulation of the KEAP1/Nrf2 signaling axis [31]. Collectively, entacapone not only inhibits the canonical target COMT but also engages multiple proteins, underscoring its considerable promise as a drug repurposing candidate.

In this study, using MST screening of an FDA-approved drug library, we identified the Parkinson’s disease drug entacapone as a specific binder of CXCR7, with an equilibrium dissociation constant Kd of 6.46 μM. Functional experiments further revealed that entacapone itself has no direct agonistic activity, but concentration dependently and subtype-selectively inhibits β-arrestin 1 recruitment induced by the endogenous ligand CXCL12 and the synthetic agonist VUF11207, while having no significant effect on β-arrestin 2 recruitment. Thus, entacapone is defined as a β-arrestin 1 biased antagonist of CXCR7.

Molecular docking predicted that entacapone interacts primarily with residues such as Trp100, Phe124, Tyr51, Phe6, and Leu305 in the transmembrane core region of CXCR7, and forms hydrogen bonds with Ser103, Asn108, Gln10, and Gln301. This compact, centrally localized binding footprint lacks substantial extension toward the extracellular vestibule, suggesting a mechanism that stabilizes the receptor in an inactive conformation and sterically obstructs the deeper insertion and multipoint binding required for agonist-induced conformational rearrangements, thereby exerting antagonistic effects.

The subtype-selective regulatory characteristics of entacapone on CXCR7 downstream signaling, specific blockade of β-arrestin 1 without affecting β-arrestin 2, offer a new pharmacological tool to understand biased signal transduction at CXCR7 [32]. Nevertheless, this study has several limitations. First, the key binding residues predicted by molecular docking (e.g., Trp100, Phe124, Gln301, and surrounding polar residues) need to be validated by site-directed mutagenesis to confirm whether they are essential for the antagonistic effect of entacapone. Second, the conservation of entacapone’s regulatory effects in primary cells or in vivo models remains to be confirmed. Third, as a β-arrestin 1-biased antagonist, the detailed effects of entacapone on downstream biological processes mediated by CXCR7 (such as receptor endocytosis, MAPK activation, and cell chemotaxis) require systematic evaluation.

Future research can be pursued in the following directions: (1) precise validation of the binding site of entacapone on CXCR7 using site-directed mutagenesis and surface plasmon resonance (SPR) to elucidate structure–activity relationships; (2) evaluation of the therapeutic potential of entacapone targeting CXCR7 in cell or animal models, inflammation, and neurodegenerative diseases to clarify its clinical translation value for repurposing; (3) optimization of affinity and selectivity through structural modification based on the nitrocatechol pharmacophore of entacapone to develop next generation CXCR7-biased modulators. The pursuit of these studies is expected to provide new strategies and candidate drugs for precision treatment of CXCR7 associated diseases.

## 4. Materials and Methods

### 4.1. Cell Culture and Materials

The 293FT and HEK293T human embryonic kidney cells were purchased from ATCC. High-glucose DMEM supplemented with 10% fetal bovine serum and 1% penicillin streptomycin was used as complete culture medium. Cell suspensions were seeded into culture flasks containing complete medium and incubated at 37 °C in a 5% CO_2_ atmosphere. When cells reached 70–80% confluence, the spent medium was aspirated, and the cells were gently washed twice with PBS. Cells were digested with 0.125% trypsin for 1 min at 37 °C, and an equal volume of complete medium was added to terminate digestion. After centrifugation, the cell suspension was subcultured at a ratio of 1:3.

Entacapone (purity ≥ 98%, T2216) was purchased from Shanghai Taoshu Biotechnology Co., Ltd. (Shanghai, China); VUF11207 (purity 99.98%, HY-100677) and FDA-Approved Drug Library (HY-L022) (https://www.medchemexpress.cn/screening/FDA-approved_Drug_Library.html, accessed on 16 July 2026) were purchased from MedChemExpress (Monmouth Junction, NJ, USA; distributed in China by MedChemExpress, Shanghai, China); Furimazine (1374040-24-0) was purchased from Yeasen Biotechnology (Shanghai, China); the Dual-Luciferase reporter assay system (E1960) was purchased from Promega Biotechnology Co., Ltd. (Beijing, China); DMEM high-glucose medium, fetal bovine serum, 0.25% trypsin-EDTA, and penicillin-streptomycin were all purchased from Gibco (Grand Island, NY, USA); Lipofectamine 3000 Transfection Reagent (L3000015), RIPA lysis and extraction buffer (89,900), and protease inhibitor (A32963) were purchased from Thermo Fisher Scientific (Waltham, MA, USA); the BCA protein assay kit (P0009) was purchased from Beyotime Biotechnology (Shanghai, China); and the MST fluorescence labeling kit and standard capillaries were purchased from NanoTemper (Munich, Germany). The pBiT2.1-CXCR7-SmBiT plasmid and the CXCR7-GFP fusion construct were generated in our laboratory, and the NanoBiT system β-arrestin 1/2 plasmids were synthesized by Sangon Biotech (Shanghai, China). The Gα_i_, Gα_s_, Gα_q_, and Gα_12_ reporter gene plasmids were kindly provided by Prof. Cheng Deng at Sichuan University.

### 4.2. MST Screening for Small Molecule Binding to CXCR7

MST was used to determine the binding affinity of ligands to CXCR7-GFP (fusion protein) or free GFP (control). In brief, 293FT cells were transfected with GFP-tagged CXCR7 or GFP only plasmids for 48 h, collected, and lysed using M-PER mammalian protein extraction reagent (Thermo Fisher Scientific) supplemented with protease/phosphatase inhibitor (Roche, Basel, Switzerland). Cell lysates were diluted in PBS to a final GFP fluorescence intensity well above the detection limit of the Monolith NT.115 instrument (NanoTemper Technologies). The ligand (10 mM in 100% DMSO) was diluted 1:100 in PBS to 100 µM (1% DMSO final), then serially diluted 1:1 in PBS with 1% DMSO to generate a 16-point series (100 µM to 3.05 nM). Ten microliters of cell lysate were mixed with 10 µL of each ligand dilution. Samples were loaded into NT.115 standard-coated capillaries (NanoTemper Technologies), and MST measurements were performed at 25 °C, 50% LED power, and medium MST power. The Kd was calculated by fitting a standard binding curve to the average of three independent dilution series. Negative controls confirmed no binding to free GFP.

For primary screening of the FDA-approved drug library (HY-L022, MedChemExpress), a “ten compounds pool” strategy was used. The 293FT cells at logarithmic growth phase were transfected with CXCR7-GFP or GFP-only plasmids for 24 h. Compounds with an MST response value greater than 7 were selected as candidates [33,34]. Secondary screening was performed using the binding affinity mode with serially diluted small molecules. Experiments were repeated three times under the same conditions. All data were analyzed using MO Affinity Analysis (×86) software (MO.Affinity Analysis Software (unlimited licenses)-NanoTemper Technologies GmbH, https://shop.nanotempertech.com/mo-affinity-analysis-software-1-license/ (accessed on 16 July 2026)).

### 4.3. Evaluation of Entacapone’s Effect on CXCR7 G Protein Signaling

Previous studies using cAMP assays, calcium flux measurements, and NanoBit experiments have demonstrated that CXCR7 does not couple with G proteins upon CXCL12 stimulation. Therefore, we employed reporter gene assays with VUF11207 as a positive control to investigate whether entacapone activates CXCR7-mediated G protein signaling.

The 293T cells were seeded into culture plates and co-transfected with the CXCR7 plasmid together with reporter gene plasmids for Gα_i_, Gα_s_, Gα_q_, or Gα_12_ pathways. After 24 h, cells were treated with increasing concentrations of entacapone, along with positive control and solvent control groups. Following drug treatment, the basal medium was removed, and cells were washed twice with room-temperature PBS. According to the manufacturer’s instructions, Bright-Glo™ luciferase assay reagent (Promega, E2610) was diluted with 1 × PBS, and 50 μL was added to each well. The plate was incubated in a cell culture incubator for 30 min to allow complete cell lysis and stabilization of the enzymatic reaction. Luminescence signals were collected using a multimode plate reader with full-wavelength detection mode and an integration time of 1 s per well, and raw RLU values were recorded. To analyze the dose dependent effect of the compound on reporter gene activity, the RLU value of each drug-treated well was divided by the mean RLU of the solvent control wells (containing an equal volume of solvent only) to obtain normalized relative luciferase activity. These values were plotted as the *Y*-axis against the logarithm of compound concentration as the *X*-axis, and dose response curves were generated using GraphPad Prism 9.0 software.

### 4.4. Evaluation of Entacapone’s Effect on CXCR7-β-Arrestin Signaling

A NanoBit-based β-arrestin recruitment assay system was established to construct a functional detection system for CXCR7. HEK293T cells were seeded into 96 well whitebottom plates and cultured until they reached 70–80% confluence. Co-transfection was performed with recombinant plasmids encoding CXCR7-SmBiT and LgBiT-β-arrestin 1/2. After 24 h, the culture medium was removed, and cells were washed twice with PBS at room temperature. The substrate furimazine was added, and the cells were incubated for 90 min at room temperature, followed by an additional 30 min static incubation. Then, gradient concentrations of CXCL12, VUF11207, and entacapone were added. Relative luminescence units (RLU) were measured using a multimode plate reader. The solvent control group only received DMSO. Data were analyzed and dose response curves were plotted using GraphPad Prism 9.0 software.

### 4.5. Binding Mode Predicted by Molecular Docking

Molecular docking was performed using AutoDock Vina 1.2.3 software following previously reported methods. The crystal structure of the CXCR7 receptor (PDB ID: 7SK6, only chain A was used) [35] was downloaded from the RCSB PDB database (https://www.rcsb.org/ accessed on 26 May 2026). Disordered, missing amino acid residues located on the distal extracellular N-terminus and extracellular loop 3 were not reconstructed via homology modeling or loop refinement, as these segments are spatially distant from the transmembrane orthosteric binding pocket and do not interfere with ligand binding prediction. Receptor preparation was completed in AutoDockTools version 1.5.7 (The Scripps Research Institute, La Jolla, CA, USA): polar hydrogens were added to the protein backbone and side chains, and Gasteiger partial charges were computed. All ionizable residues were assigned physiological protonation states at pH 7.4: aspartate/glutamate side chains were deprotonated, and lysine/arginine side chains were fully protonated. To validate the docking protocol, self-redocking was carried out with the native co-crystallized ligand extracted from the 7SK6 complex using the same preparation and docking parameters as described below. The optimal redocked conformation was aligned to the experimental crystal ligand, yielding a heavy-atom RMSD of 1.405 (VUF11207-CXCR7) and 1.496 (Entacapone-CXCR7), which confirmed the reliability of our grid and parameter setup for reproducing the native binding pose of CXCR7. The three dimensional structures of entacapone (PubChem CID: 5281081) and the positive control ligand VUF11207 (PubChem CID: 59052285) were retrieved from the PubChem database, optimized with the MMFF94 force field, and then processed in AutoDockTools by adding hydrogens, assigning charges, and setting rotatable bonds, and finally exported as PDBQT files. A grid box was centered on the transmembrane active pocket of CXCR7 (coordinates were adjusted based on pocket residues; box dimensions: 40 Å × 40 Å × 40 Å, spacing: 0.375 Å). Conformational searches were performed using the BFGS algorithm with 20 docking runs; all other parameters were kept at default values. After docking, the conformation with the lowest binding energy was selected. Interactions between the ligands and key receptor residues, including hydrogen bonds and hydrophobic interactions, were analyzed using PyMOL version 2.4 to evaluate the stability and specificity of the binding modes.

## 5. Conclusions

This study demonstrates that entacapone, a clinically approved COMT inhibitor, specifically binds to CXCR7 and acts as a β-arrestin 1-biased antagonist. Entacapone concentration dependently and subtype-selectively inhibits CXCL12 and VUF11207 induced β-arrestin 1 recruitment, without affecting β-arrestin 2. Molecular docking suggests that entacapone interacts with key residues such as Trp100, Phe124, and Tyr51 in the transmembrane domain, stabilizing the receptor in an inactive conformation. These findings provide a new pharmacological tool for studying CXCR7 signaling and support the repurposing of entacapone for CXCR7-associated diseases, including cancer, inflammation, and autoimmune disorders.

## Figures and Tables

**Table 1:** Detailed molecular interactions of entacapone and VUF11207 with CXCR7.

Chemicals	Binding Energy (kcal/mol)	Interaction Type	Binding Sites	Distance (Å)
Entacapone	−9.79	Hydrophobic Interaction	Phe6	2.96
			Glu10	3.47
			Trp100	3.21
			Phe124	3.14
			Gln301	3.29
			Leu305	2.56
		Hydrogen Bonds	Glu10	2.91
			Tyr51	3.12
			Ser103	1.96
			Asn108	2.21
VUF11207	−13.45	Hydrophobic Interaction	His4	2.89
			Leu5	3.61
			Trp100	3.57
			Phe124	3.73
			Phe129	3.43
			Val272	3.93
		Hydrogen Bond	Gln301	2.56
		Halogen Bond	Glu213	2.31

## Data Availability

All data supporting the results of this study are included in the manuscript, and the datasets are available upon request.

## Abbreviations

The following abbreviations are used in this manuscript:

CXCR7	C-X-C chemokine receptor type 7
ACKR3	Atypical chemokine receptor 3
COMT	Catechol-O-methyltransferase
CXCL12	C-X-C motif chemokine ligand 12
GFP	Green fluorescent protein
GPCR	G protein-coupled receptor
GRK	G protein-coupled receptor kinase
MAPK	Mitogen-activated protein kinase
MST	Microscale thermophoresis
NanoBit	NanoLuc binary technology
PD	Parkinson’s disease
S/N	Signal-to-noise ratio
RLU	Relative luminescence units
RIPA	Radioimmunoprecipitation assay buffer
FDA	Food and Drug Administration
EMT	Epithelial–mesenchymal transition
ERK	Extracellular signal-regulated kinase
GSK3β	Glycogen synthase kinase 3 beta
ROS	Reactive oxygen species
ECL2	Extracellular loop 2
